# Supplementary material for: Quantitative modeling of human liver reveals dysregulation of glycosphingolipid pathways in nonalcoholic fatty liver disease
Source: iScience. 2022 Aug 15;25(9):104949. doi: 10.1016/j.isci.2022.104949 (PMC9440293; doi:10.1016/j.isci.2022.104949)
Supplement: Document S1. Figures S1–S17 and Tables S1–S4 [file mmc1.pdf]

iScience, Volume *25*

Supplemental information

Quantitative modeling of human liver reveals dysregulation of glycosphingolipid pathways in nonalcoholic fatty liver disease

Partho Sen, Olivier Govaere, Tim Sinioja, Aidan McGlinchey, Dawei Geng, Vlad Ratziu, Elisabetta Bugianesi, Jörn M. Schattenberg, Antonio Vidal-Puig, Michael Allison, Simon Cockell, Ann K. Daly, Tuulia Hyötyläinen, Quentin M. Anstee, and Matej Orešič

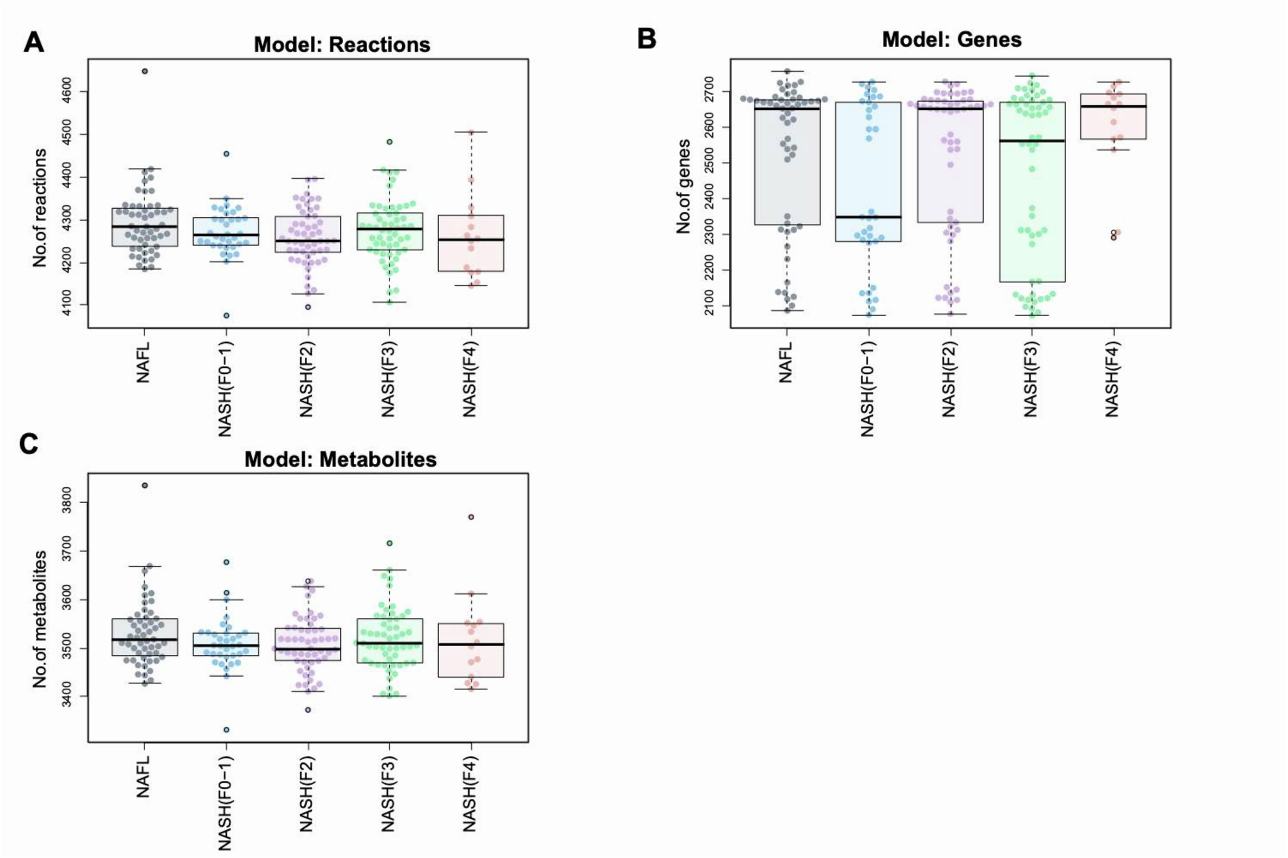

Figure S1. Personalized genome-scale metabolic models of human hepatocytes in NAFLD.

Boxplots showing distribution of number of reactions (**A**), genes (**B**) and metabolites (**C**) (marked by dots) contained in the genome-scale metabolic models (GEMs) of human hepatocytes developed for (n=206) subjects and contextualized for various stages of NAFLD using transcriptomics data. Related to **Figure 1**.

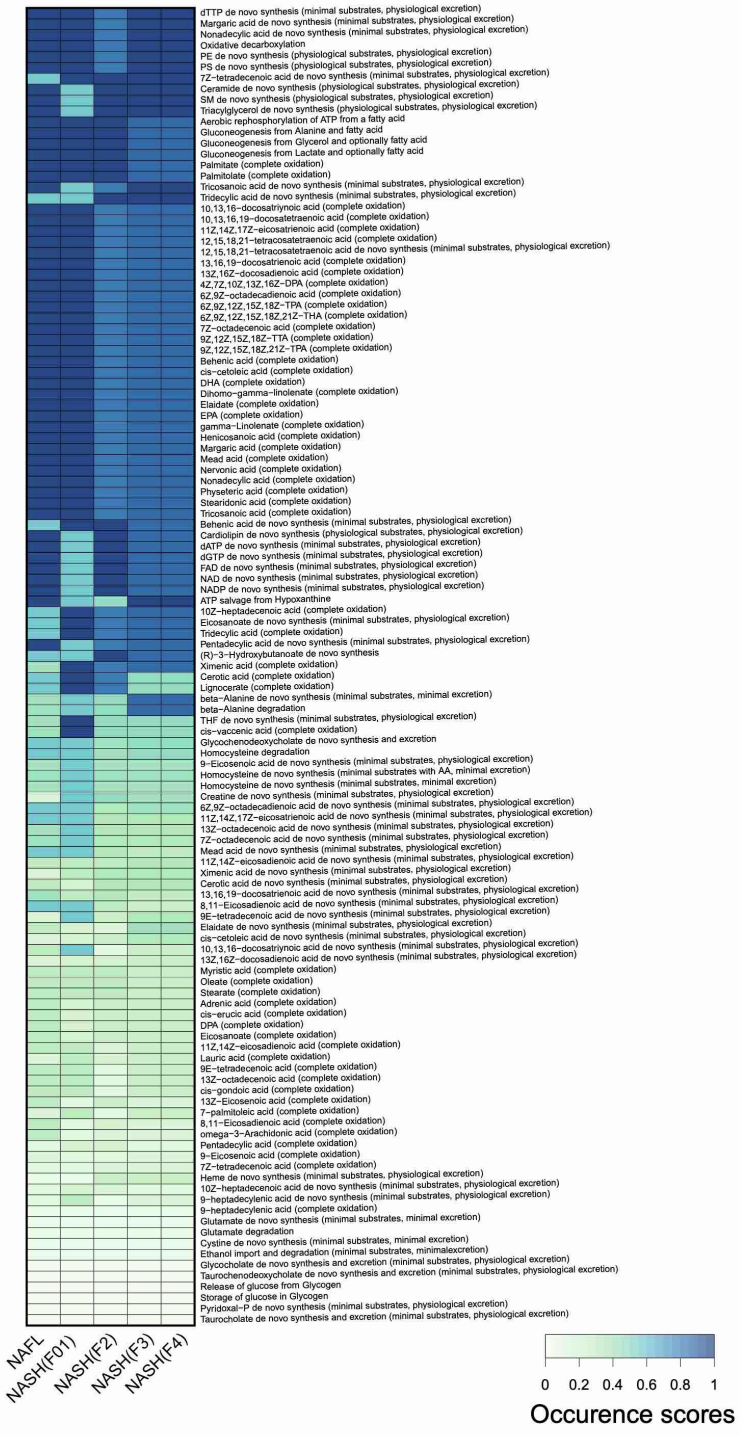

Figure S2. Metabolic tasks carried out by the NAFLD stage-specific personalized GEMs.

[0-1] denotes low to high occurrence scores (OS). OS acquired by a GEM is based on its capability (‘Yes’ or ‘No’) to perform the assigned metabolic task. Related to **Figures 1** and **2**.

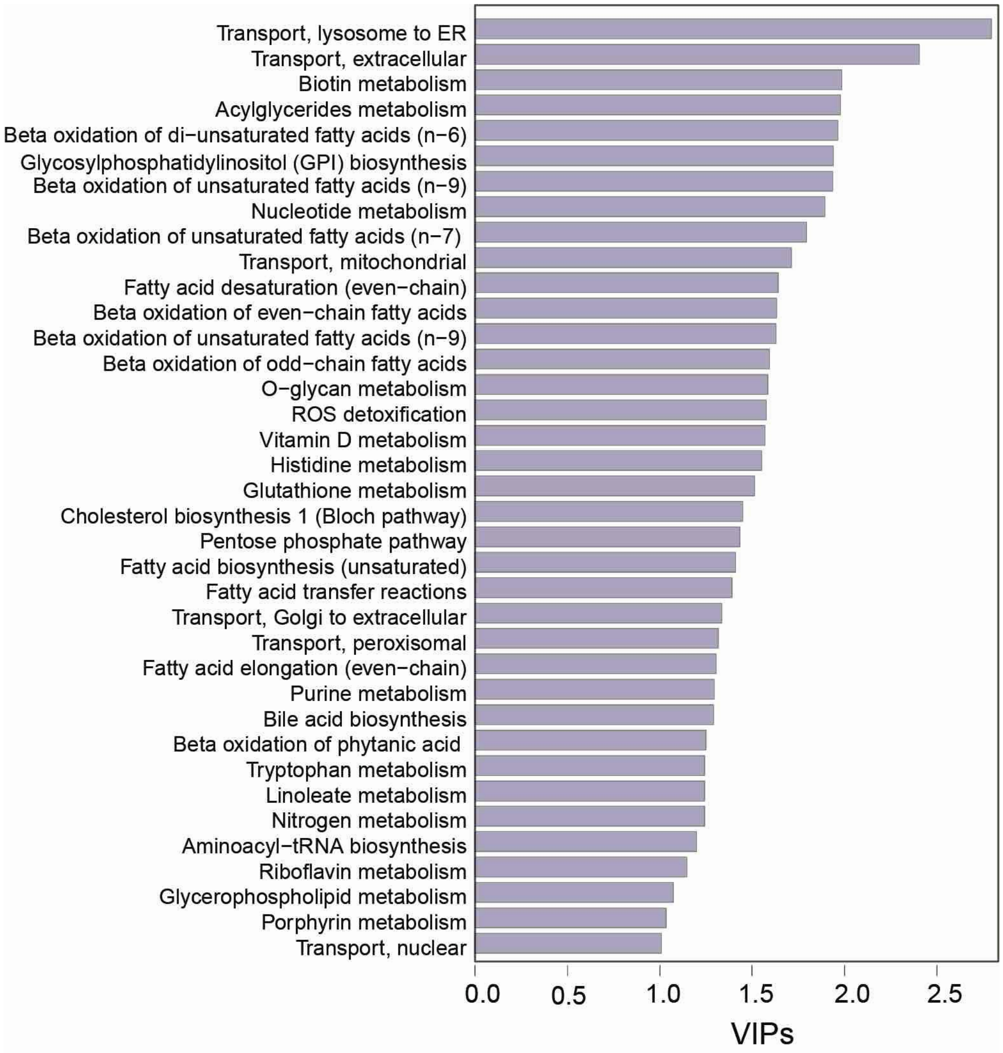

Figure S3. Metabolic pathways/subsystems with (PLS-DA, VIP score >1). Related to **Figure 2**.

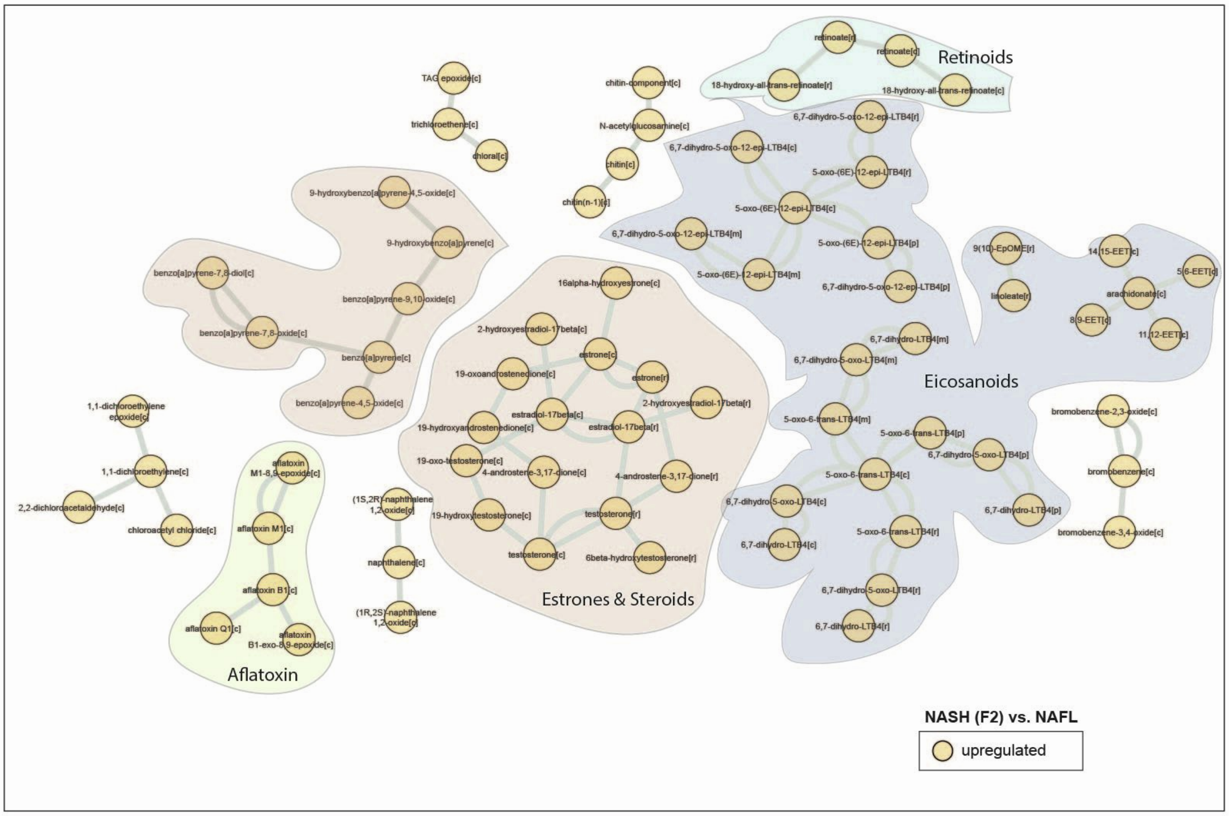

Figure S4. A metabolic-centric view of reporter metabolite (RM) modules that were significantly altered between NASH F2 *vs.* NAFL.

Orange color denotes upregulated (p<0.05 adjusted for FDR). Each node represents a 'RM' and single or double lines represent reversible or irreversible metabolic reactions respectively. RMs that belong to a particular chemical class are color coded. Related to **Figure 3**.

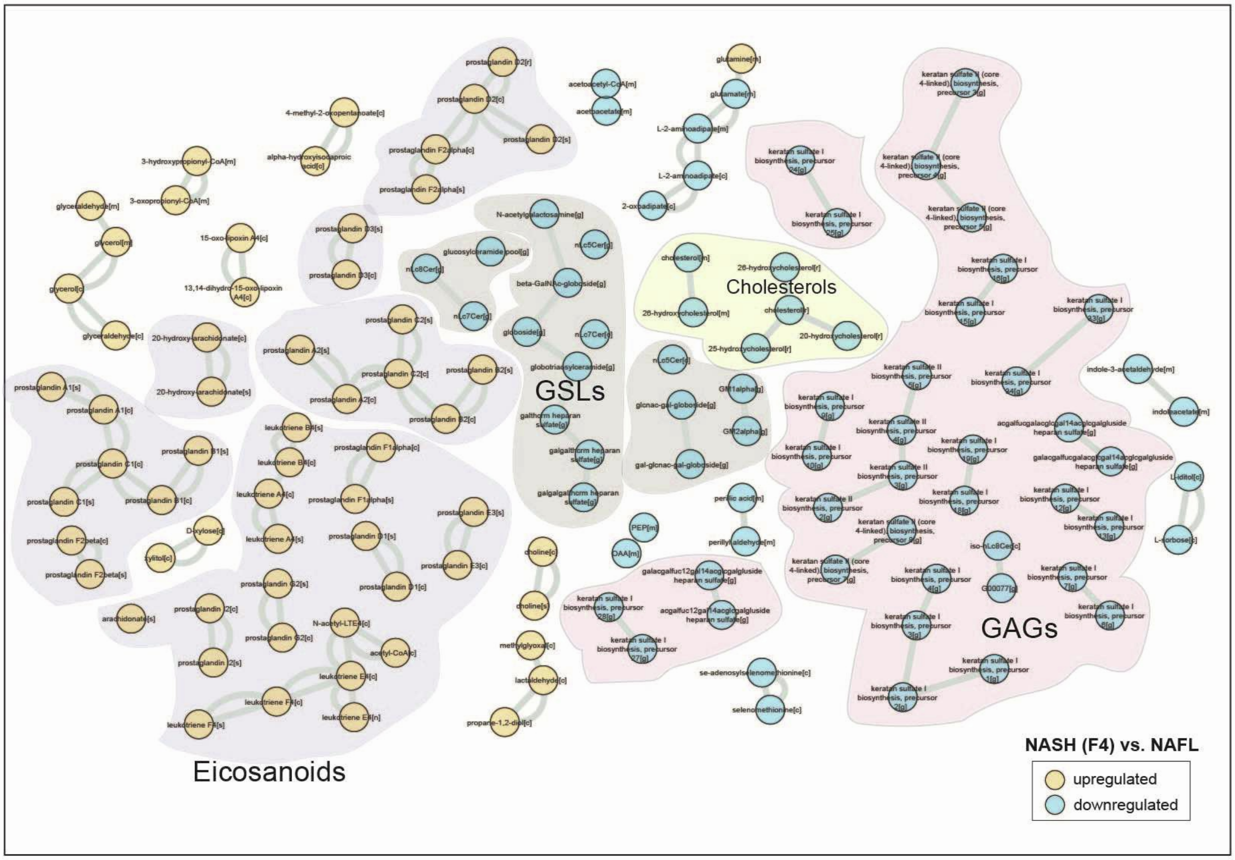

Figure S5. A metabolic-centric view of reporter metabolite (RM) modules that were significantly altered between NASH F4 *vs.* NAFL.

Orange and blue color denotes up- and downregulated ($p<0.05$ adjusted for FDR) respectively. Each node represents a 'RM' and single or double lines represent reversible or irreversible metabolic reactions respectively. RMs that belong to a particular chemical class are color coded. Related to **Figure 3.**

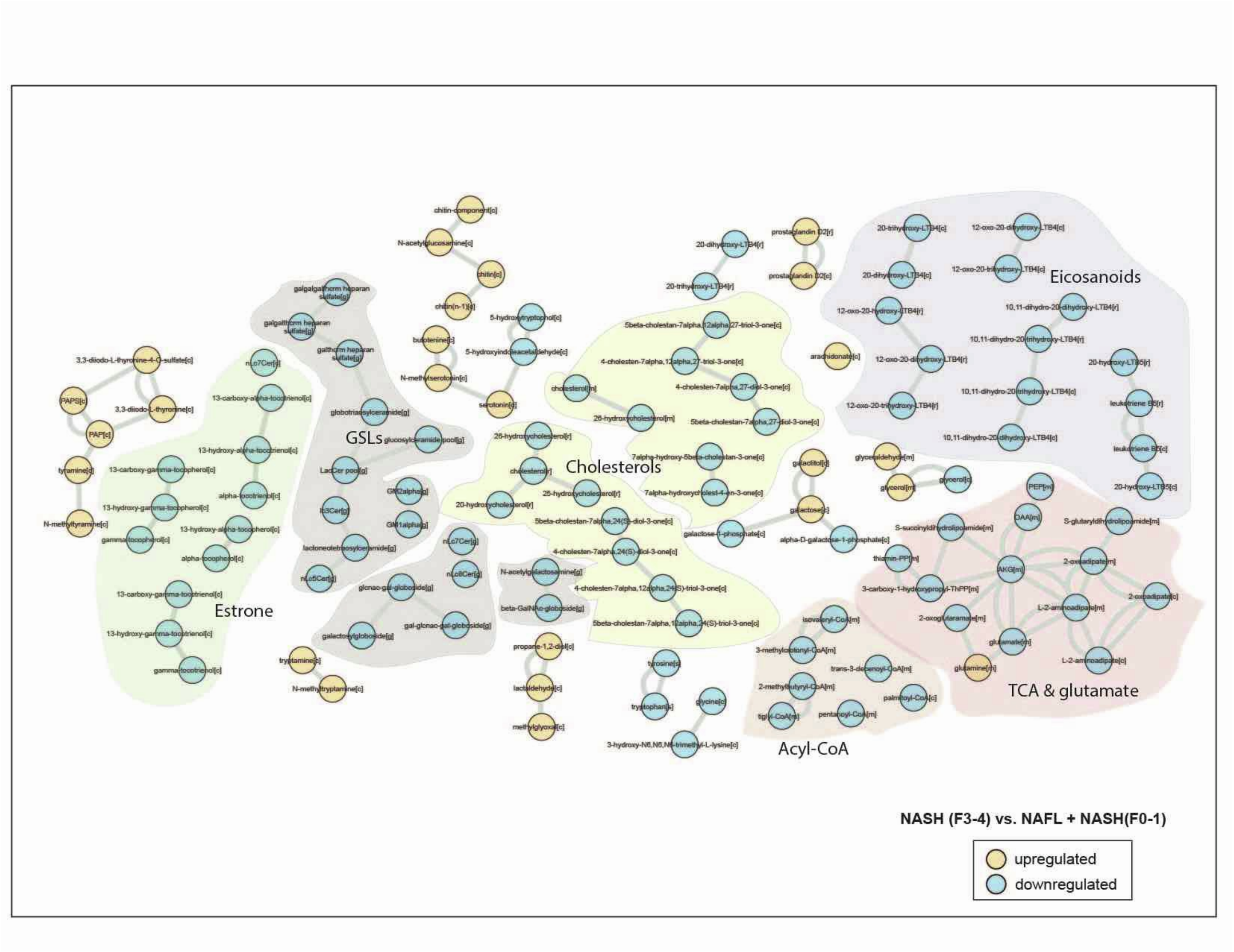

Figure S6. A metabolic-centric view of reporter metabolite (RM) modules that were significantly altered between ‘advanced’ fibrosis *vs.* ‘minimum’ disease.

Orange and blue color denotes up and downregulated ($p<0.05$ adjusted for FDR) respectively. Each node represents a ‘RM’ and single or double lines represent reversible or irreversible metabolic reactions respectively. RMs that belong to a particular chemical class are color coded. Related to **Figure 3**.

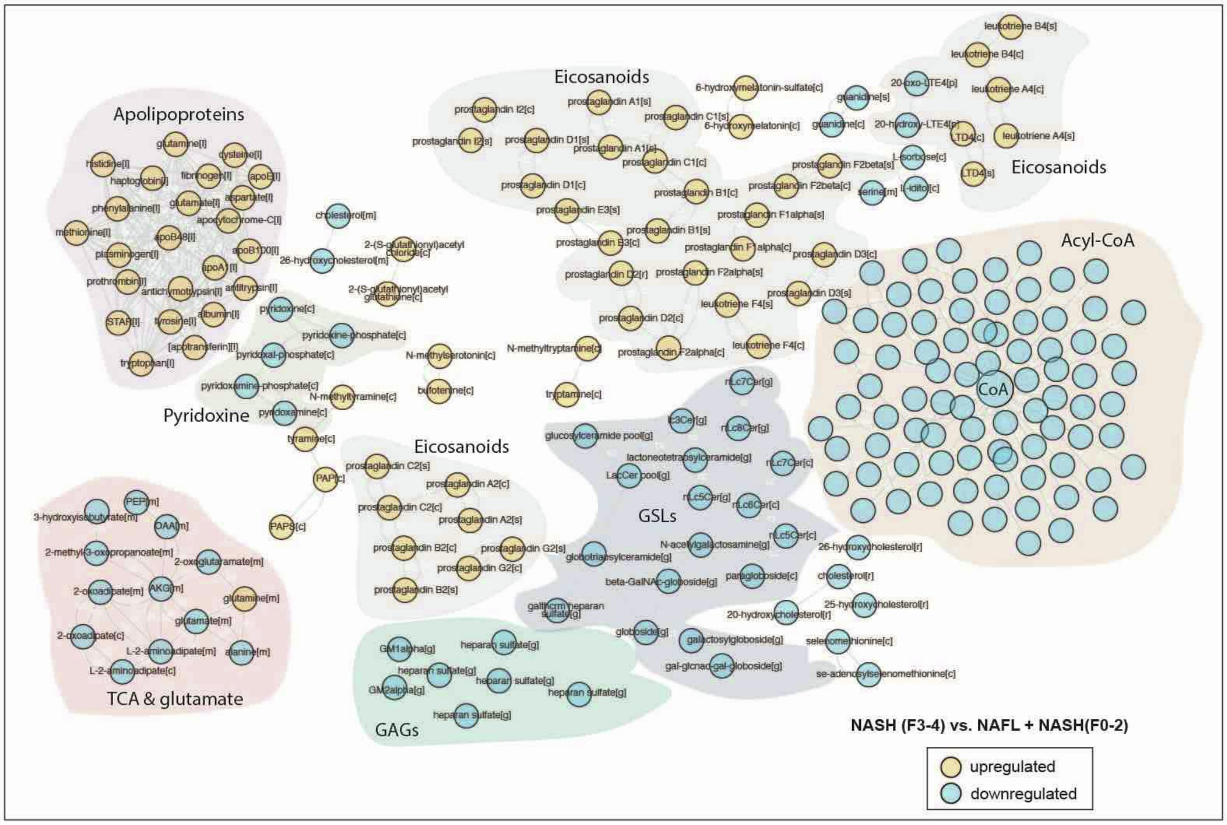

Figure S7. A metabolic-centric view of reporter metabolite (RM) modules that were significantly altered between ‘advanced’ fibrosis *vs.* ‘mild disease.

Orange and blue color denotes up and downregulated (p<0.05 adjusted for FDR) respectively. Each node represents a ‘RM’ and single or double lines represent reversible or irreversible metabolic reactions respectively. RMs that belong to a particular chemical class are color coded. Related to **Figure 3**.

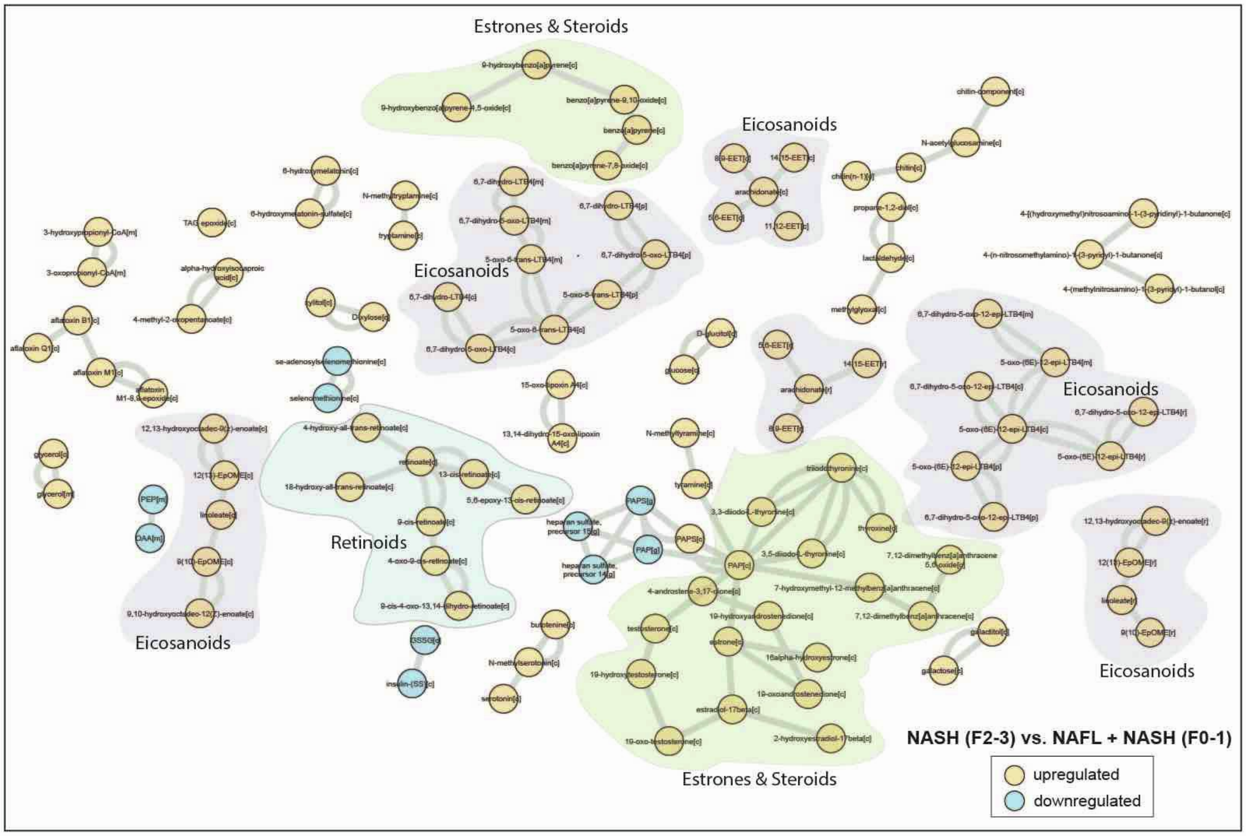

Figure S8. A metabolic-centric view of reporter metabolite (RM) modules that were significantly altered between 'clinically significant' non-cirrhotic fibrosis *vs.* 'minimum' disease.

Orange and blue color denotes up and downregulated ($p<0.05$ adjusted for FDR) respectively. Each node represents a 'RM' and single or double lines represent reversible or irreversible metabolic reactions respectively. RMs that belong to a particular chemical class are color coded. Related to **Figure 3**.

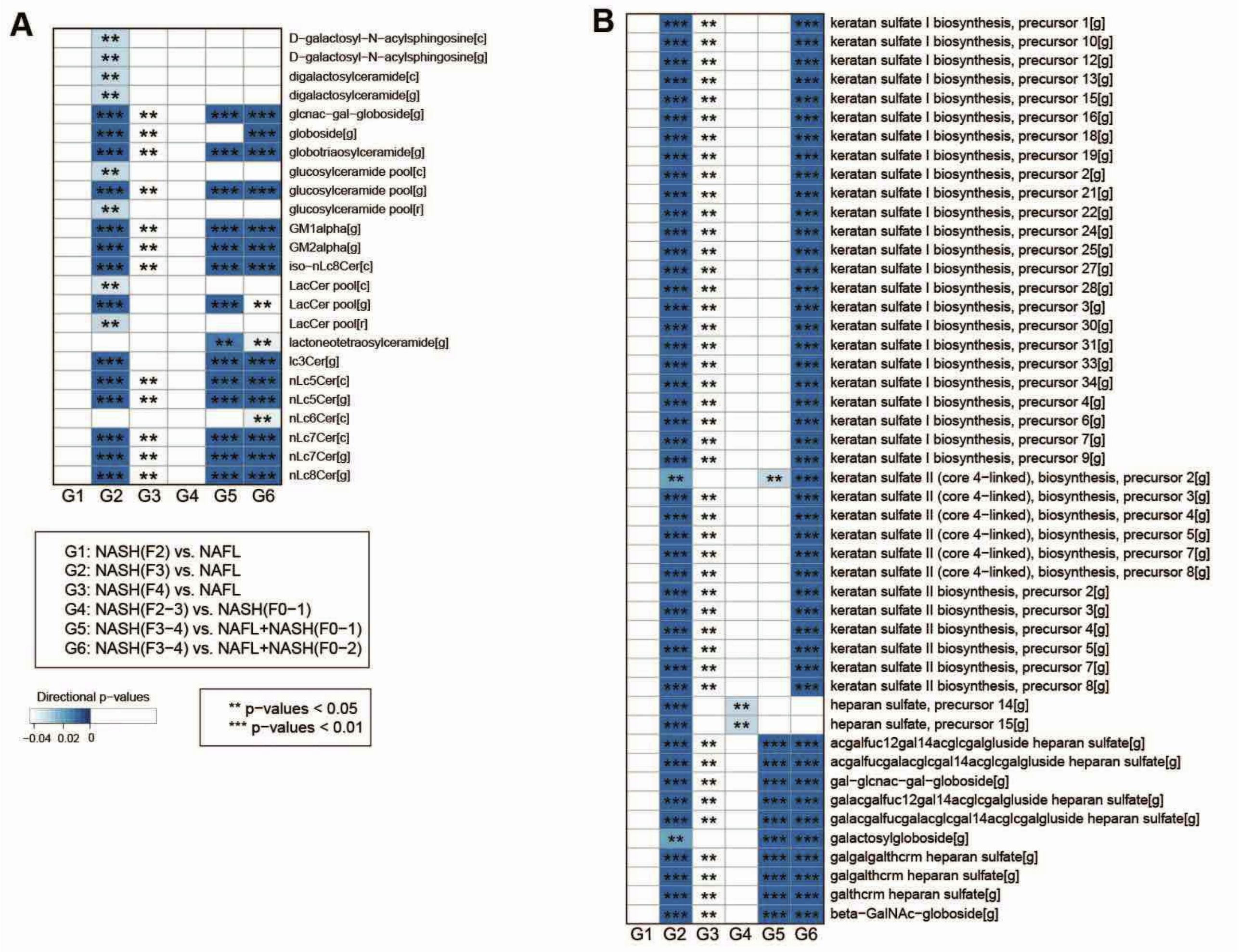

Figure S9. Heatmap showing RM clusters of (**A**) glycosphingolipids (GSLs) and (**B**) glycosaminoglycans (GAGs) that are significantly (***p<0.01, **p<0.05, adjusted for FDR) downregulated (blue color) along the different stages NAFLD. Related to **Figure 3**.

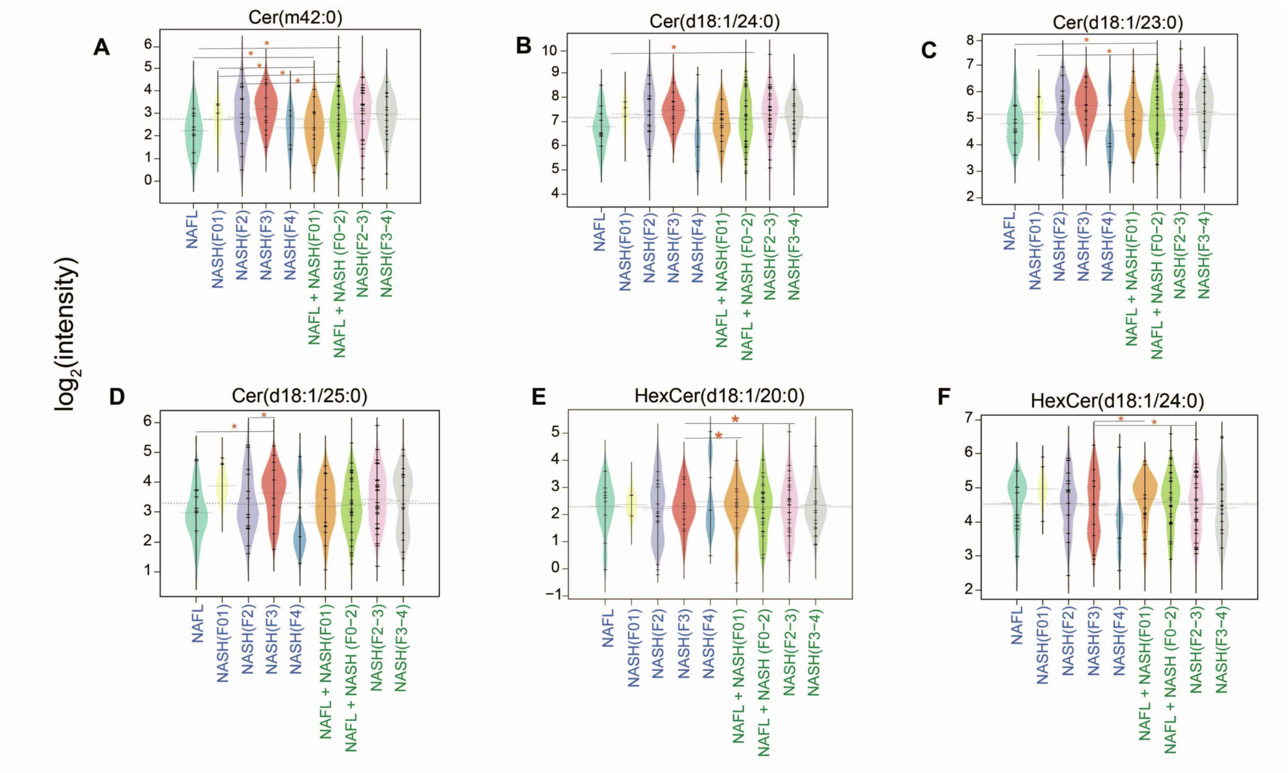

Figure S10. Serum metabolic levels of ceramides and glycosphingolipids in the patients (n=41) at different stages of NAFLD.

(**A-F**) Beanplots showing the $\log_2$ intensities of the Cers and GSLs across different stages of NAFLD. The dotted line denotes the mean of the population, and the black dashed lines in the bean plots represent the group mean. Related to **Figure 5**.

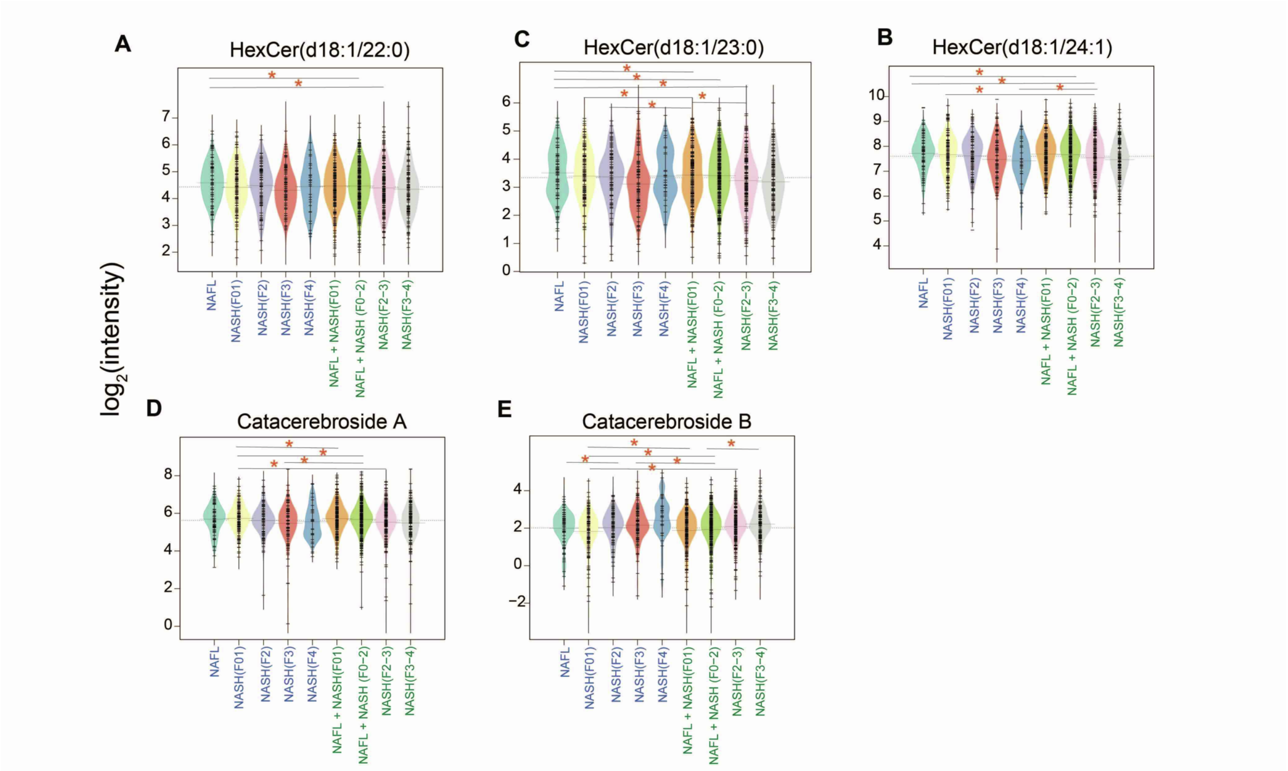

Figure S11. Serum metabolic levels of glycosphingolipids in the patients (n=360) at different stages of NAFLD.

(**A-E**) Beanplots showing the $\log_2$ intensities of GSLs across different stages of NAFLD. The dotted line denotes the mean of the population, and the black dashed lines in the bean plots represent the group mean. Related to **Figure 5**.

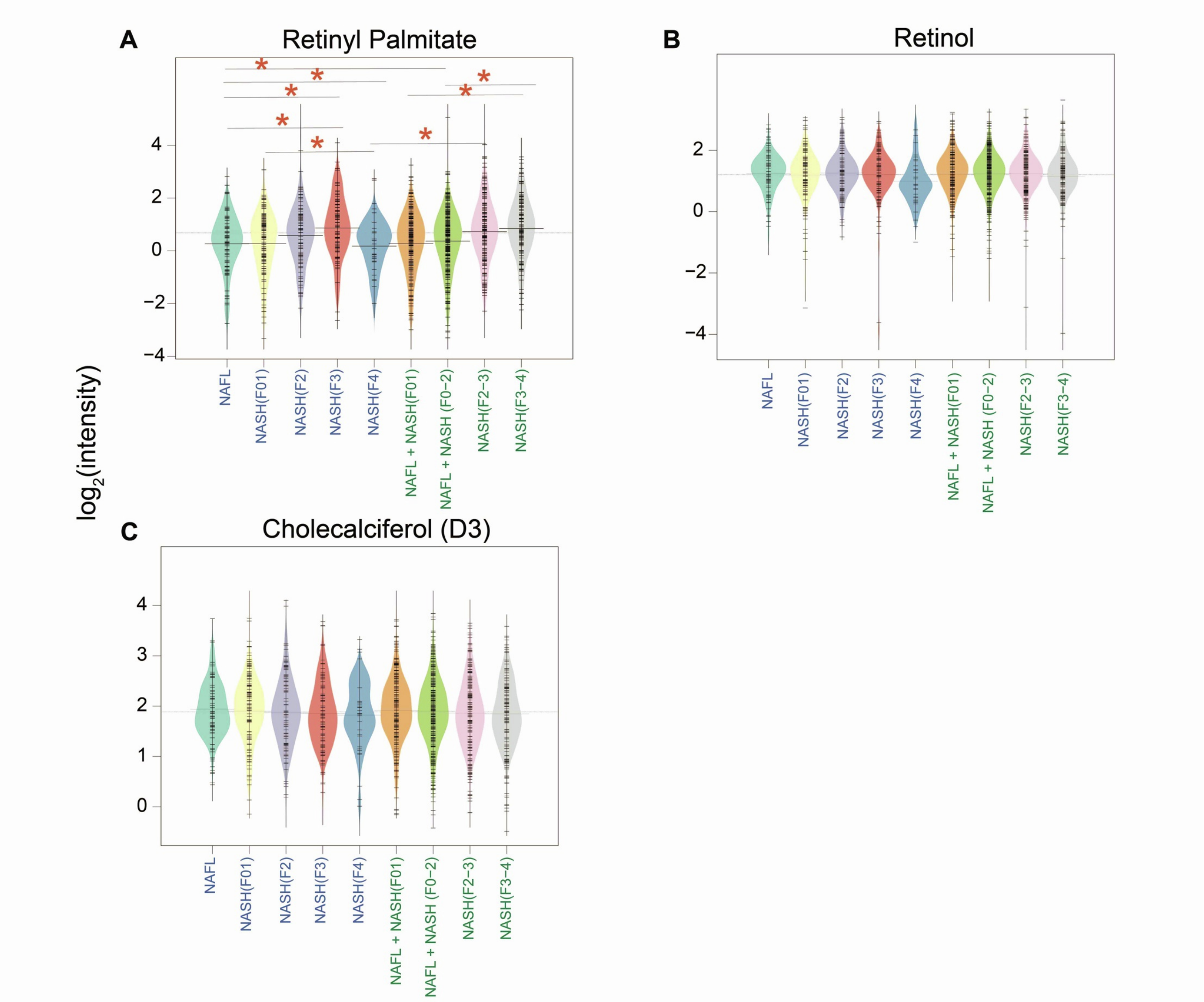

Figure S12. Serum levels of retinoids in the patients (n=360) at different stages of NAFLD. (**A-C**) Beanplots showing the $\log_2$ intensities of the retinoids across different stages of NAFLD. The dotted line denotes the mean of the population, and the black dashed lines in the bean plots represent the group mean. Related to **Figure 6**.

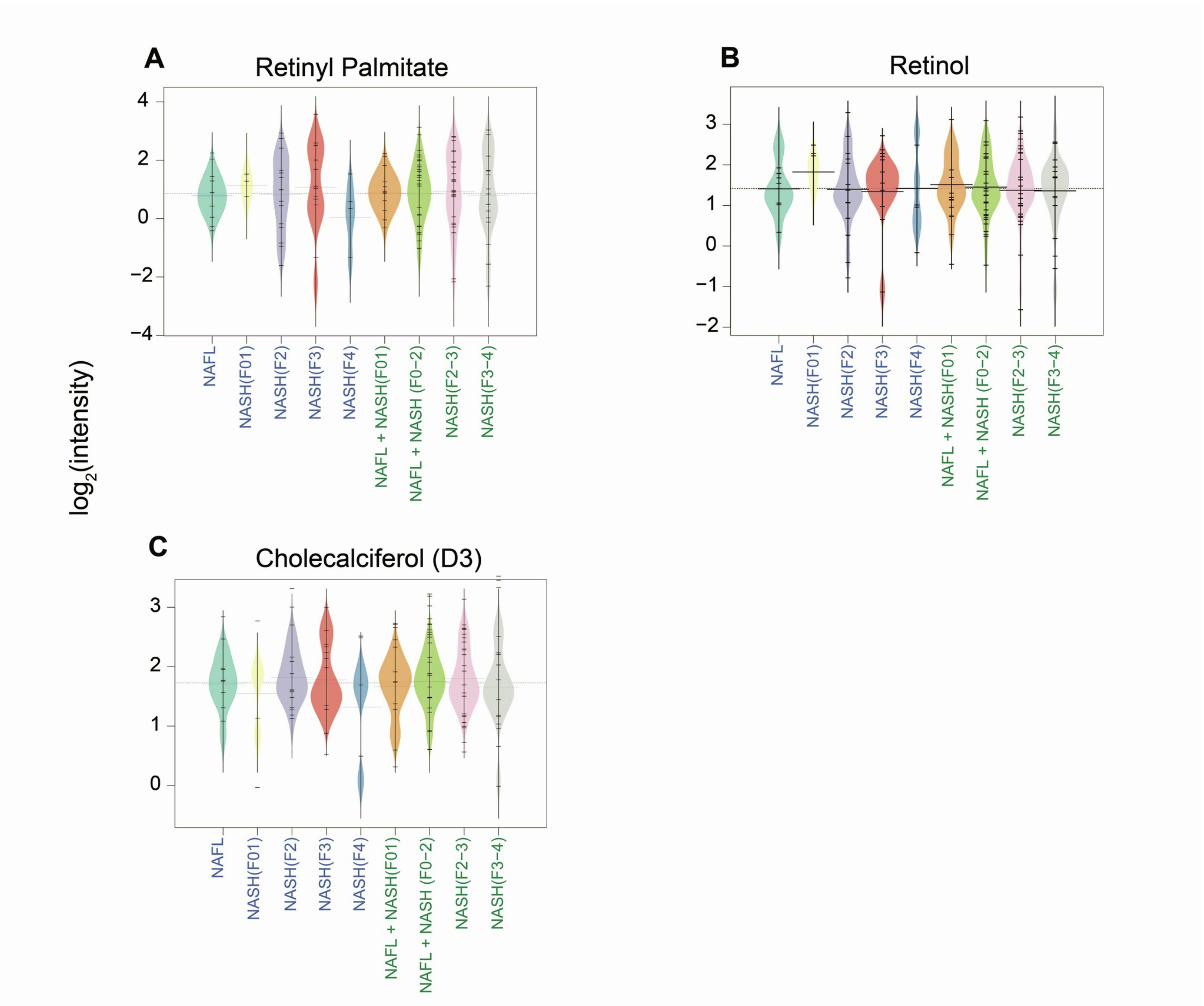

Figure S13. Serum levels of retinoids in the patients (n=41) at different stages of NAFLD. (**A-C**) Beanplots showing the $\log_2$ intensities of the retinoids across different stages of NAFLD. The dotted line denotes the mean of the population, and the black dashed lines in the bean plots represent the group mean. Related to **Figure 6**.

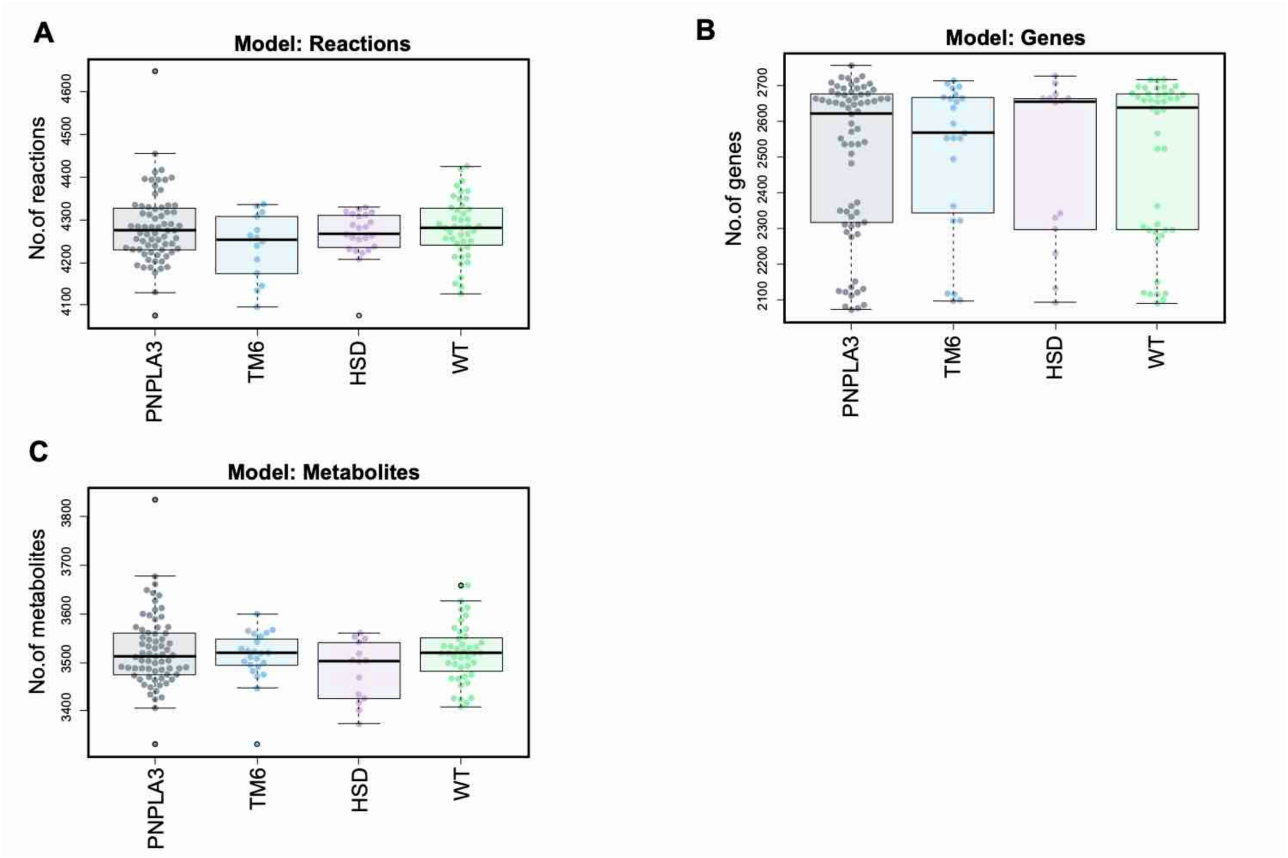

Figure S14. Personalized genome-scale metabolic models of human hepatocytes contextualized for gene variants and WT.

Boxplots showing distribution of number of reactions (**A**), genes (**B**) and metabolites (**C**) (marked by dots) contained in the genome-scale metabolic models (GEMs) of human hepatocytes developed for (n=139) subjects, and contextualized for three major genetic variants (*PNPLA3, TM6SF2* and *HSD17B13*) and WT; associated with risk and severity of NAFLD. 'WT' is a wild-type model. Related to **Figure 6**.

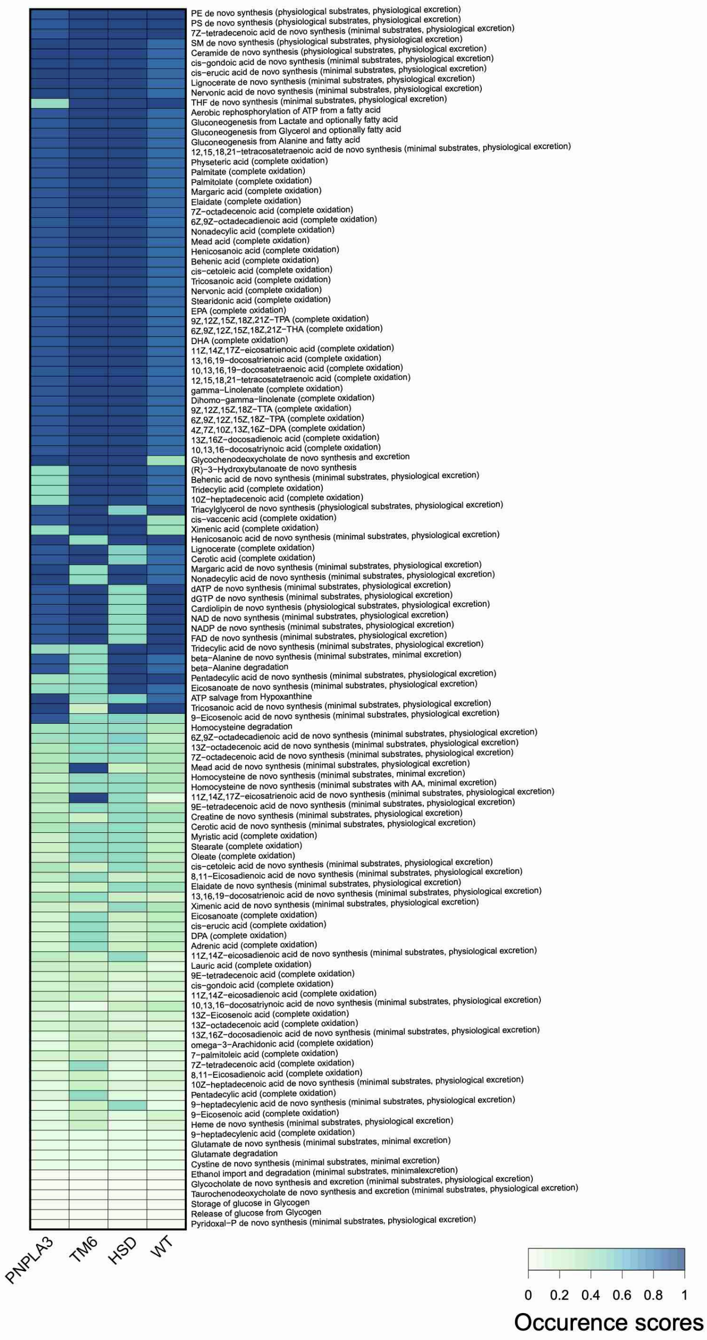

Figure S15. Metabolic tasks carried out by the personalized GEMs contextualized for the gene variants and wild type (WT).

[0-1] denotes low to high occurrence scores (OS). OS acquired by a GEM is based on its capability ('Yes' or 'No') to perform the assigned metabolic task. Related to **Figure 6**.

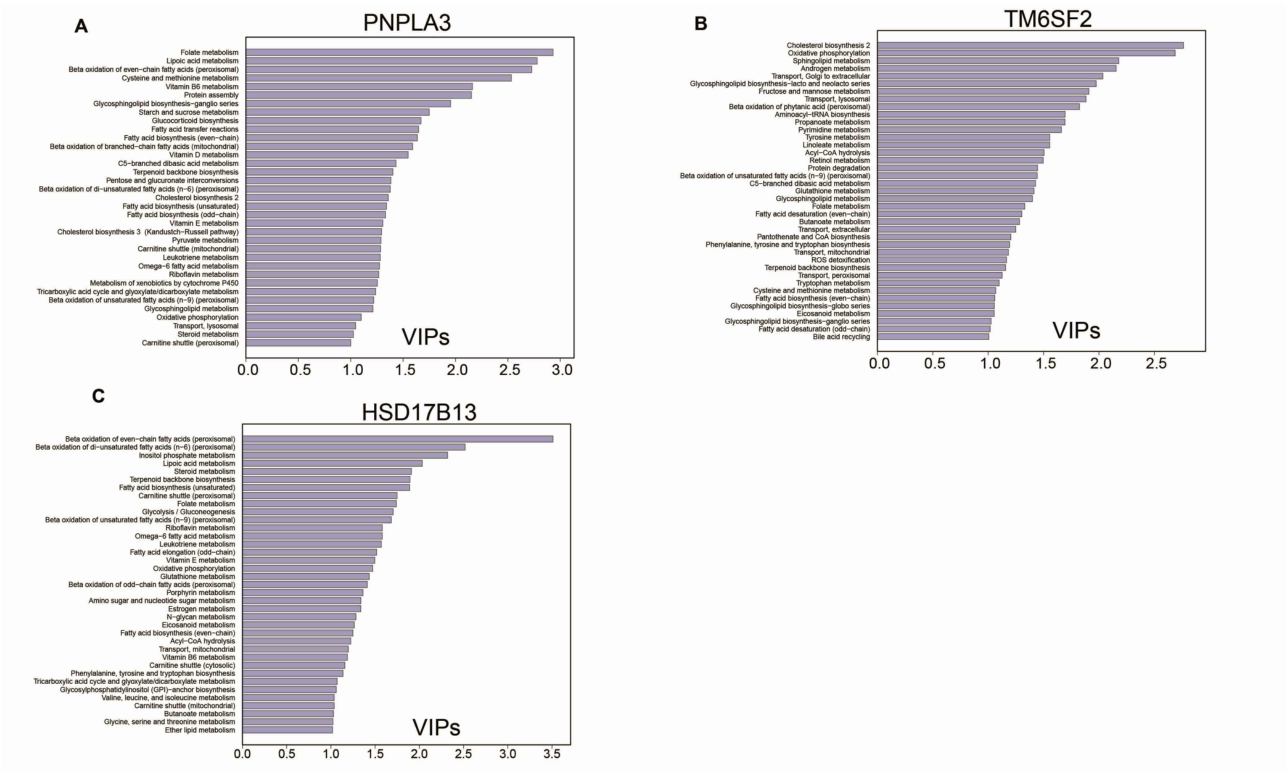

Figure S16. Variable Importance in Projection (VIP) scores derived from PLS-DA of the metabolic subsystems/pathways differences across three major gene variants vs. WT groups. (**A-C**) Metabolic subsystems sorted by their (VIP scores >1) retrieved by fitting pairwise PLS-DA models for (**A**) *PNPLA3* (**B**) *TM6SF2* and (**C**) *HSD17B13* gene variants vs. WT. Related to **Figure 6**.

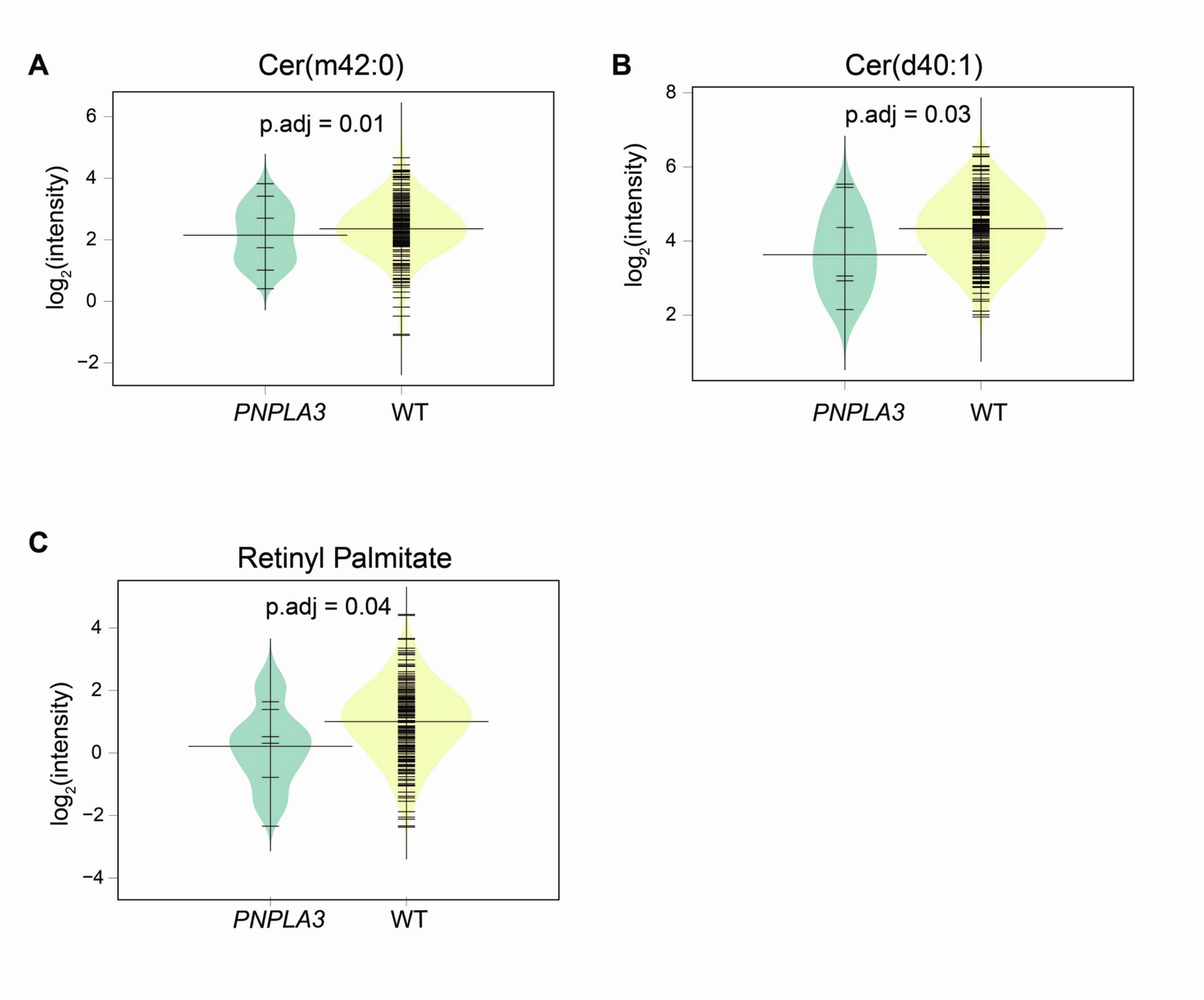

Figure S17. Difference in the intensities of metabolites between the *PNPLA3* variant vs. WT groups. (**A-C**) Beanplots showing differences in the $\log_2$ intensities of the Cer(m42:0), Cer(d40:1) and retinyl palmitate measured in the serum samples of a patient subgroup exclusively carrying variants for *PNPLA3* (homo- and heterozygous) gene vs. WT. The dotted line denotes the mean of the population, and the black dashed lines in the bean plots represent the group mean. Statistical significance was determined by (two-sample t-test, $p<0.05$ adjusted for FDR, p.adj). Related to **Figure 6**.

Table S1. Demographic characteristics of the diesease groups. Related to **Figures 2–4**.

**Clinical features**	**NAFL** (n=51)	**NASH (F0-1)** (n=34)	**NASH (F2)** (n=53)	**NASH (F3)** (n=54)	**NASH (F4)** (n=14)
Age (mean ± SD)	50.55 ± 13.73	51.32 ± 11.89	53.23 ± 11.69	57.81 ± 9.06	61.21 ± 8.42
Sex					
male	35	23	27	31	7
female	16	11	26	23	7
BMI (mean ± SD)	29.42 ± 4.14	28.44 ± 8.96	31.11 ± 8.04	31.34 ± 6.28	30.01 ± 13.84
T2DM					
no	38	20	23	12	3
yes	13	14	30	41	11
ALT (mean ± SD)	50.37 ± 33.39	65.91 ± 33.87	75.66 ± 56.18	73.17 ± 35.72	65.86 ± 33.77
AST (mean ± SD)	34.43 ± 15.48	30.79 ± 12.34	48.53 ± 27.86	51.98 ± 26.51	50.29 ± 27.40

Table S2. Demographic characteristics of the Type 2 Diabetes (T2DM) vs no T2DM groups. Related to **Figures 2–4**.

		**T2DM yes** (n=110)	**T2DM no** (n=96)
Sex	male	61	62
	female	49	34
Age		57.04 +/- 10.21	50.51 +/- 12.7
BMI		31.58 +/- 6.44	30.41 +/- 5.13
ALT		63.21 +/- 35.08	71.61 +/- 47.81
AST		45.70 +/- 22.78	43.47 +/- 23.49
HBA1C		56.26 +/- 14.92	38.69 +/- 5.90
Anti-hyperglycemic drugs	no	26	87
	yes	74	2
	--	10	7
Grade of Steatosis	0	0	0
	1	25	35
	2	49	24
	3	36	37
Hepatocyte Ballooning	0	14	38
	1	57	41
	2	39	17
Kleiner Lobular inflammation	0	7	9
	1	47	48
	2	44	36
	3	12	3
SAF Lobular inflammation	0	7	9
	1	72	68
	2	62	19
NAS>=4	yes	87	63
SAF activity >=2	yes	95	59
Stage Kleiner (5-tier staging system)	0	11	27
	1	16	31
	2	30	23
	3	42	12
	4	11	3

Table S3. Selection of individuals carrying variants for *PNPLA3*, *TM6SF2* and *HSD17B13* genes associated with risk and severity of NAFLD. Related to **Figure 6**.

**Gene variants**	**RNA-seq cohort** (Govaere et al., 2020) **(Individuals)**	**No. of participants**
PNPLA3 rs738409 (CC/GC/GG)	206	**69** individuals exclusively exhibiting carriers of *PNPLA3 rs738409* (GG, GC) and homozygous / wild types for *TM6SF2 rs58542926* (CC) and *HSD17B13 rs72613567* (--).
PNPLA3 rs738409 (CC)	75	
PNPLA3 rs738409 (GG)	42	
PNPLA3 rs738409 (GC)	89	
TM6SF2 rs58542926 (CC/CT/TT)	206	**13** individuals exclusively exhibiting carriers of *TM6SF2 rs58542926* (CT, TT) and homozygous / wild types for *PNPLA3 rs73840* (CC) and *HSD17B13 rs72613567* (--).
TM6SF2 rs58542926 (CC)	156	
TM6SF2 rs58542926 (CT)	2	
TM6SF2 rs58542926 (TT)	48	
HSD17B13 rs72613567 (--/-T/TT)	188	**21** individuals exclusively exhibiting carriers of *HSD17B13 rs72613567* (-T, TT) and homozygous / wild types for *TM6SF2 rs58542926* (CC) and *PNPLA3 rs73840* (CC).
HSD17B13 rs72613567 (--)	120	
HSD17B13 rs72613567 (TT)	7	
HSD17B13 rs738409 (-T)	61	
Wild types, WTs (*PNPLA3 rs738409 (CC) + TM6SF2 rs58542926 (CC) + HSD17B13 rs72613567 (--)*)	--	**36** individual wild types for *PNPLA3 rs738409, TM6SF2 rs58542926 and HSD17B13 rs72613567*)

Table S4. Assignment of (n=206) individuals exclusively carrying variants (homo- and heterozygous) for *PNPLA3*, *TM6SF2* and *HSD17B13* to their respective NAFLD groups. Related to **Figure 6**.

**Gene variants**	**NAFL** (n)	**NASH (F0-1)** (n)	**NASH (F2)** (n)	**NASH (F3)** (n)	**NASH (F4)** (n)
PNPLA3	17	14	16	17	5
TM6SF2	2	1	5	3	2
HSD17B13	8	4	2	7	0
WT	11	5	9	8	3
